# Parallel comparison of fibroblast-like synoviocytes from the surgically removed hyperplastic synovial tissues of rheumatoid arthritis and osteoarthritis patients

**DOI:** 10.1186/s12891-019-2977-2

**Published:** 2019-12-07

**Authors:** Wei Huang, Linlin Zhang, Chao Cheng, Wenshan Shan, Ruixiang Ma, Zongsheng Yin, Chen Zhu

**Affiliations:** 1 Department of Orthopaedics, The First Affiliated Hospital of USTC, Division of Life Sciences and Medicine, University of Science and Technology of China, Hefei, Anhui 230001 People’s Republic of China; 2 Department of Orthopaedics, The First Affiliated Hospital of Anhui Medical University, Hefei, Anhui 230022 People’s Republic of China

**Keywords:** OA, RA, FLS, Isolation, Characterization

## Abstract

**Background:**

Fibroblast-like synoviocytes (FLS) are essential cellular components in inflammatory joint diseases such as osteoarthritis (OA) and rheumatoid arthritis (RA). Despite the growing use of FLS isolated from OA and RA patients, a detailed functional and parallel comparison of FLS from these two types of arthritis has not been performed.

**Methods:**

In the present study, FLS were isolated from surgically removed synovial tissues from twenty-two patients with OA and RA to evaluate their basic cellular functions.

**Results:**

Pure populations of FLS were isolated by a sorting strategy based on stringent marker expression (CD45^−^CD31^−^CD146^−^CD235a^−^CD90^+^PDPN^+^). OA FLS and RA FLS at the same passage (P2-P4) exhibited uniform fibroblast morphology. OA FLS and RA FLS expressed a similar profile of cell surface antigens, including the fibroblast markers VCAM1 and ICAM1. RA FLS showed a more sensitive inflammatory status than OA FLS with regard to proliferation, migration, apoptosis, inflammatory gene expression and pro-inflammatory cytokine secretion. In addition, the responses of OA FLS and RA FLS to both the pro-inflammatory cytokine tumor necrosis factor-alpha (TNF-α) and the anti-inflammatory drug methotrexate (MTX) were also evaluated here.

**Conclusion:**

The parallel comparison of OA FLS and RA FLS lays a foundation in preparation for when FLS are considered a potential therapeutic anti-inflammatory target for OA and RA.

## Background

Rheumatoid arthritis (RA) is a chronic autoimmune disease, and the inflammatory synovium is full of hyperplastic fibroblast-like synoviocytes (FLS) [1]. Moreover, many patients with osteoarthritis (OA) also develop low-grade synovitis, and there is growing evidence that synovitis affects symptoms and the rate of cartilage degeneration in patients with OA. Synovial infiltration by FLS and other immune cells has been demonstrated in the different stages of OA [2].

FLS stem from the mesenchymal lineage and exhibit aggressive and invasive cellular characteristics, secreting a range of cytokines and matrix factors that recruit other immune cells to the inflammatory synovium, eventually leading to cartilage injury and bone erosion [3–5]. Therefore, FLS are targets for disease-modifying drugs and anti-inflammatory drugs, which has resulted in a substantial body of work characterizing these cells and examining their contribution to human disease using conventional methods of isolation, such as differential plating of dissected tissues or magnetic bead isolation [6, 7]. However, the low efficiency of these methods and the lack of knowledge regarding parallel comparison of OA FLS and RA FLS hinders research utilizing these cells in vitro to delineate the pathology of different arthritis joint diseases.

The aims of this study were to comprehensively characterize and compare the cellular function and inflammatory phenotype of FLS from the synovium of OA and RA patients by using a stringent sorting method. In particular, we were interested in investigating the proliferation, migration, apoptosis, and expression and production of inflammatory markers as well as in determining how this inflammatory phenotype is maintained over prolonged cell culture (P2-P4) and in response to pro-inflammatory cytokines and anti-inflammatory drugs.

## Methods

### Clinical specimens

Synovial tissues were obtained from OA and RA patients (all Chinese) upon joint replacement or synovectomy at the First Affiliated Hospital of USTC and the First Affiliated Hospital of Anhui Medical University, Hefei, China. This study was approved by the Ethics Committee of the First Affiliated Hospital of USTC and the First Affiliated Hospital of Anhui Medical University. The informed consent was signed by each of the patients and their guardians. All RA patients (*n* = 23) include in this study were diagnosed with RA according to the 2010 American College of Rheumatology/European League Against Rheumatism (ACR/EULAR) criteria [8]. The diagnosis of primary knee OA (*n* = 23) conformed to the criteria from the American College of Rheumatology (ACR) [9], radiographic changes were grade III or IV according to the Kellgren and Lawrence classification method [10]. RA and OA patients induced by trauma, inflammation, tuberculosis, or sepsis were excluded.

### Isolation and in vitro culture of cells from OA and RA synovia

The method used for the isolation of FLS from synovial tissues was modified from a method previously described [11]. The synovium was removed in joints of patients with OA and RA. Removed hyperplastic synovial tissues were washed and minced in DMEM (Life Technologies, Gaithersburg, MD, USA) supplemented with 10% FBS (Gibco, Grand Island, USA), penicillin and streptomycin (Gibco, Grand Island, USA). Dissected synovial tissues were digested in culture media containing 1 mg/ml collagenase IV (Sigma, St. Louis, MO, USA) and 0.1 mg/ml deoxyribonuclease I (Sigma, St. Louis, MO, USA), and then it was incubated for 2 h at 37 °C. Tissues were then vortexed and resuspended in fresh media. The media containing the digested tissues was centrifuged, and the cell pellet was resuspended in fresh media for cell sorting. The sorted OA FLS and RA FLS were cultured at 37 °C in 5% CO_2_. Culture media were replenished every 2 days, and cells were subcultured when they reached 90% confluence.

### Flow cytometry

OA and RA FLS were incubated with fluorescein isothiocyanate (FITC)-, phycoerythrin (PE)- or allophycocyanin (APC)-conjugated antibodies at 37 °C for 1 h: CD45-FITC (5 μg/test), CD31-FITC (5 μg/test), CD146-FITC (5 μg/test), CD235a-FITC (5 μg/test), PDPN-PE (2 μg/test), CD90-APC (2 μg/test), CD106 (VCAM1)-FITC (5 μg/test), CD54 (ICAM1)-PE (2 μg/test). All flow antibodies were from Miltenyi Biotechnology Company (Bergisch Gladbach, Germany). After 3 times of washing by pre-cooled PBS, the cells were analysed by flow cytometer (BD FACSAria II) and BD FACSDiva software. For each test, 2 × 10^8^ total synovial cells from RA and OA synovium were used. It’s around (1.3 ± 0.4) × 10^8^ and (1.0 ± 0.3) × 10^8^ total count (events) in gate P4 for RA samples and OA samples. Isotype controls were included in the FACS detection for those proteins with relatively low expression (IgG1-FTIC for CD146, and REA control-FITC for CD31 and CD235a, Additional file 1: Figure S1). Unlabelled cells were used in other FACS assays as negative controls.

### H&E staining, Masson staining and immunohistochemistry (IHC)

OA and RA synovial tissues were fixed with acetone for 15 min, washed twice with PBS, and then incubated for 1 h in a humid chamber with primary antibodies (PCNA, Santa Cruz Biotec, Santa Cruz, CA, USA; VCAM-1, Santa Cruz Biotec; ICAM-1, Santa Cruz Biotec). The synovial tissues were then washed 3 times with PBS and incubated for an additional hour with an isotype-matched horseradish peroxidase (HRP)-conjugated secondary antibody (anti-rat IgG, Santa Cruz Biotec). After 3 additional washes, the HRP reaction was developed with diaminobenzidine (DAB) per the manufacturer’s instructions.

Paraffin-embedded OA and RA synovial tissues were sliced and then blocked with 5% goat serum for 30 min. The tissues were incubated in primary anti-PCNA (Santa Cruz Biotec), anti-CD90 (Santa Cruz Biotec), anti-ICAM1 (Abcam, Cambridge, UK) or anti-VCAM1 (Abcam, Cambridge, UK) antibodies overnight at 4 °C. IHC staining was generally performed as previously reported. The primary antibody was omitted in negative controls. H&E staining and Masson staining were performed according to standard protocols.

### Proliferation

The proliferation abilities of OA and RA FLS were evaluated by CCK-8 assay (Bestbio, Nanjing, China) and Ki-67 staining (Miltenyi Biotec). All procedures were performed according to standard protocols.

### Migration

Transwell (Costar) cell culture plates were used for migration and invasion assays. A total of 5 × 10^4^ FLS were seeded into the upper chamber in serum-free medium, and serum-containing medium was added into the lower wells. After 24 h or 48 h, the upper chamber was washed with PBS twice and fixed with 4% paraformaldehyde for 20 min, and then 1% crystal violet was used to stain the invasive cells.

### Apoptosis

Annexin V/propidium iodide (PI) staining was performed for the detection of apoptotic cells. After the desired treatment, 1 × 10^6^ cells were collected and washed twice with ice-cold PBS. The cells were then stained using the Alexa Fluor®488 Annexin V/Dead Cell Apoptosis Kit with Alexa Fluor 488 annexin V and PI (Miltenyi Biotec) for flow cytometry according to the manufacturer’s guidelines. The untreated cells served as a negative control for double staining.

### Western blot

OA and RA FLS were lysed in SDS buffer. The protein concentrations were determined via a BCA kit (Thermo Fisher Scientific, Waltham, MA, USA). Equal amounts of cell lysates were run on a gel, transferred onto a PVDF membrane (Millipore, Billerica, MA, USA), and probed with the following primary antibodies: interleukin-1 beta (IL-1β) (1:200, Santa Cruz Biotec), interleukin-6 (IL-6) (1:500, Santa Cruz Biotec) and GAPDH (1:4000, Abcam). GAPDH served as a loading control. After incubation with secondary antibodies, signals were visualized by enhanced chemiluminescence (GE system).

### Elisa

A total of 2 × 10^5^ cells were seeded in a 12-well plate for 48 h. Supernatants were collected to measure the amounts of secreted IL-6 and TNF-α. IL-6 and TNF-α were detected via a human IL-6 ELISA kit and a human TNF-α ELISA kit (Miltenyi Biotec), respectively.

### qRT-PCR

Cultures of OA and RA FLS were grown to confluence in 6-well culture plates. Cells were treated for 24 h with TNF-α (20 ng/ml) or Methotrexate (MTX, 100 μM). Twelve hours after stimulation, whole-cell RNA was collected by TRIzol (Life Technologies), and it was analysed by qRT-PCR for gene expression. GAPDH was used as an endogenous control. A reverse transcription kit and qRT-PCR dye were obtained from Takara Biomedical Technology Company (Shiga, Japan).

### Statistics

The error bars mean ± Standard Error of Mean (SEM) of six independent experiments. *, ** and *** indicate *P* < 0.05, *P* < 0.01 and *P* < 0.0001, respectively (Student’s t-test).

## Results

**Fig. 1:**
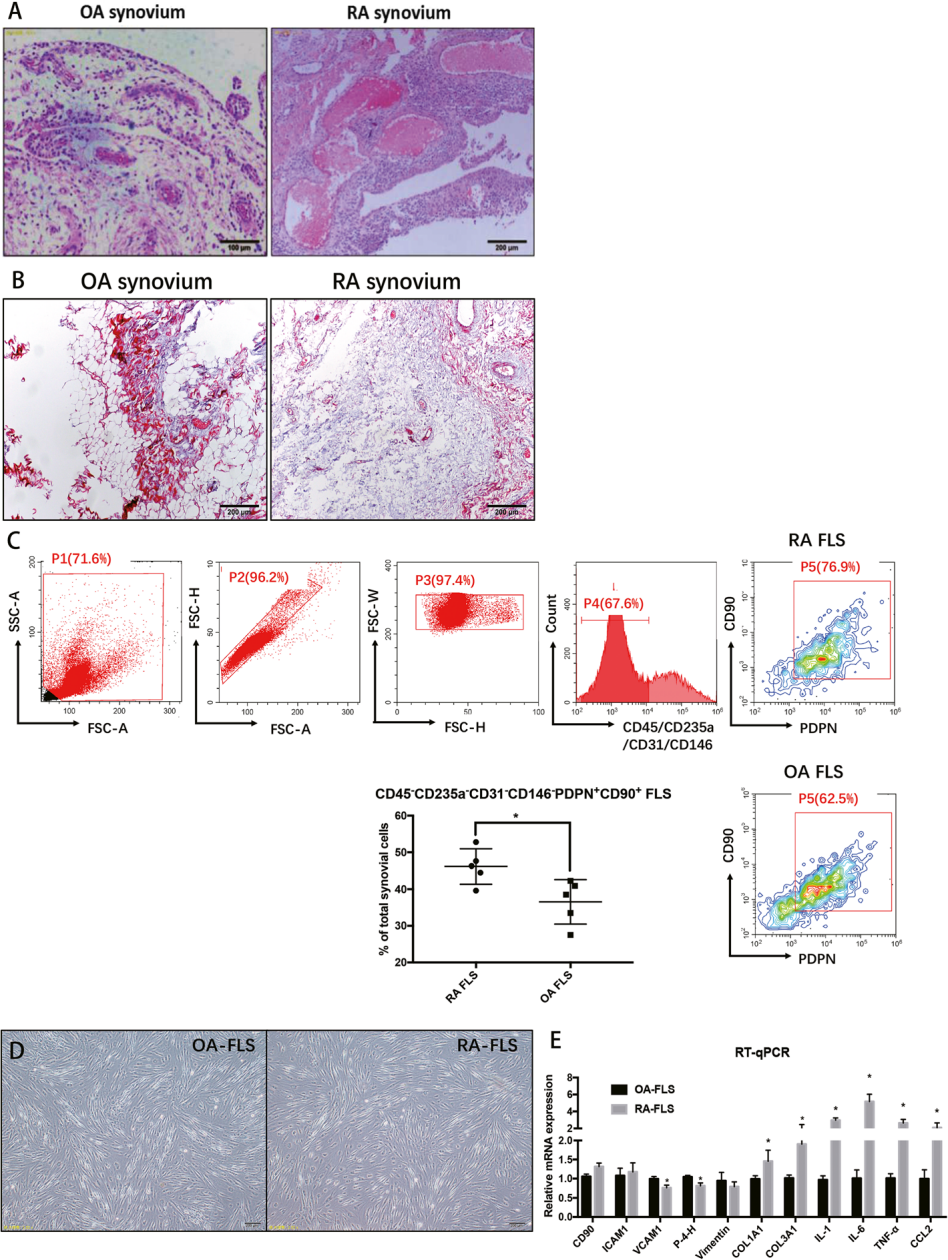
**a** H&E staining showed the typical pathological status of OA and RA hyperplastic synovial tissues. **b** Masson staining validated that the hyperplastic fibroblast is more prominent in an RA synovium. **c** The sorted number of FLS using the CD45^−^CD31^−^CD146^−^CD235a^−^CD90^+^PDPN^+^ sorting strategy. **d** The sorted OA and RA FLS exhibited a classic spindle-shaped fibroblastic morphology. **e** The comparison of markers of FLS in both OA FLS and RA FLS, as measured by qRT-PCR. Each bar in the figure represents the mean ± SEM of triplicates. *P < 0.05

**Fig. 2:**
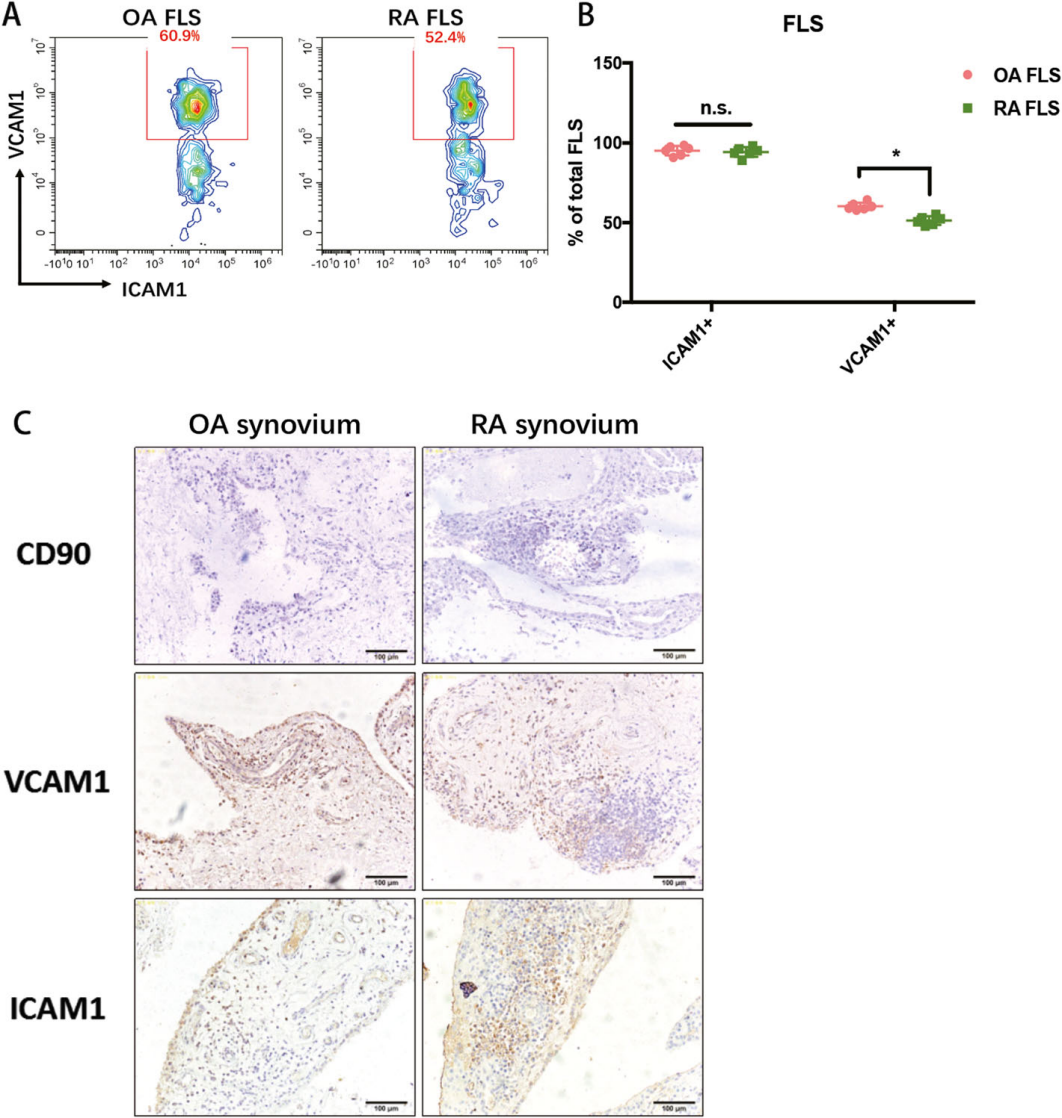
**a** FACS analysis of ICAM1 and VCAM1 expression in OA FLS and RA FLS. **b** Calculation of ICAM1 and VCAM1 expression in OA FLS and RA FLS. **c** IHC analysis of CD90, ICAM1 and VCAM1 expression in OA and RA synovium. Each bar in the figure represents the mean ± SEM of six experiments. *P < 0.05

Twenty-three joint synovium biopsies from OA and RA patients were used in this experiment. H&E staining showed the typical pathological status of OA and RA hyperplastic synovial tissues (Fig. 1a). Masson staining validated that hyperplastic fibroblasts are more prominent in an RA synovium than an OA synovium (Fig. 1b). To isolate homogenous FLS from RA and OA synovial tissues, we readily identified CD45^−^CD31^−^CD146^−^CD235a^−^CD90^+^PDPN^+^ FLS [12] after collagenase/ deoxyribonuclease digests of patient tissues. The ratio of isolated RA FLS in total synovial cells was significantly higher than that of OA FLS (Fig. 1c, RA FLS 46.5% ± 6.9% and OA FLS 37.2% ± 8.6%), suggesting that the hyperplasticity in the synovium of RA patients is at least partially due to FLS hyper-proliferation. For characterization the cells, only those that actively proliferated from passage 2 to passage 4 were used. Both OA and RA FLS showed a typical spindle shape and formed clusters at confluency (Fig. 1d). In addition, qRT-PCR was performed to compare some common fibroblast markers in OA FLS and RA FLS (Fig. 1e), including Prolyl-4-hydroxylase (P-4-H), Vimentin, procollagen I (COL1A1) and procollagen III (COL3A1). Among them, the expression of P-4-H and Vimentin were lower in RA FLS than in OA FLS, and the expression of procollagen I and procollagen III were up-regulated in RA FLS compared to that in OA FLS. After short-term in vitro culture, ICAM1 was found by FACS analysis to be expressed in nearly all OA and RA FLS (Fig. 2a and b), while VCAM1 expression was much higher in OA FLS (~ 60.9%) than in RA FLS (~ 52.4%) (Fig. 2a and b), which was validated by the IHC staining for CD90, ICAM1 and VCAM1 in OA and RA synovial tissues (Fig. 2c).

**Fig. 3:**
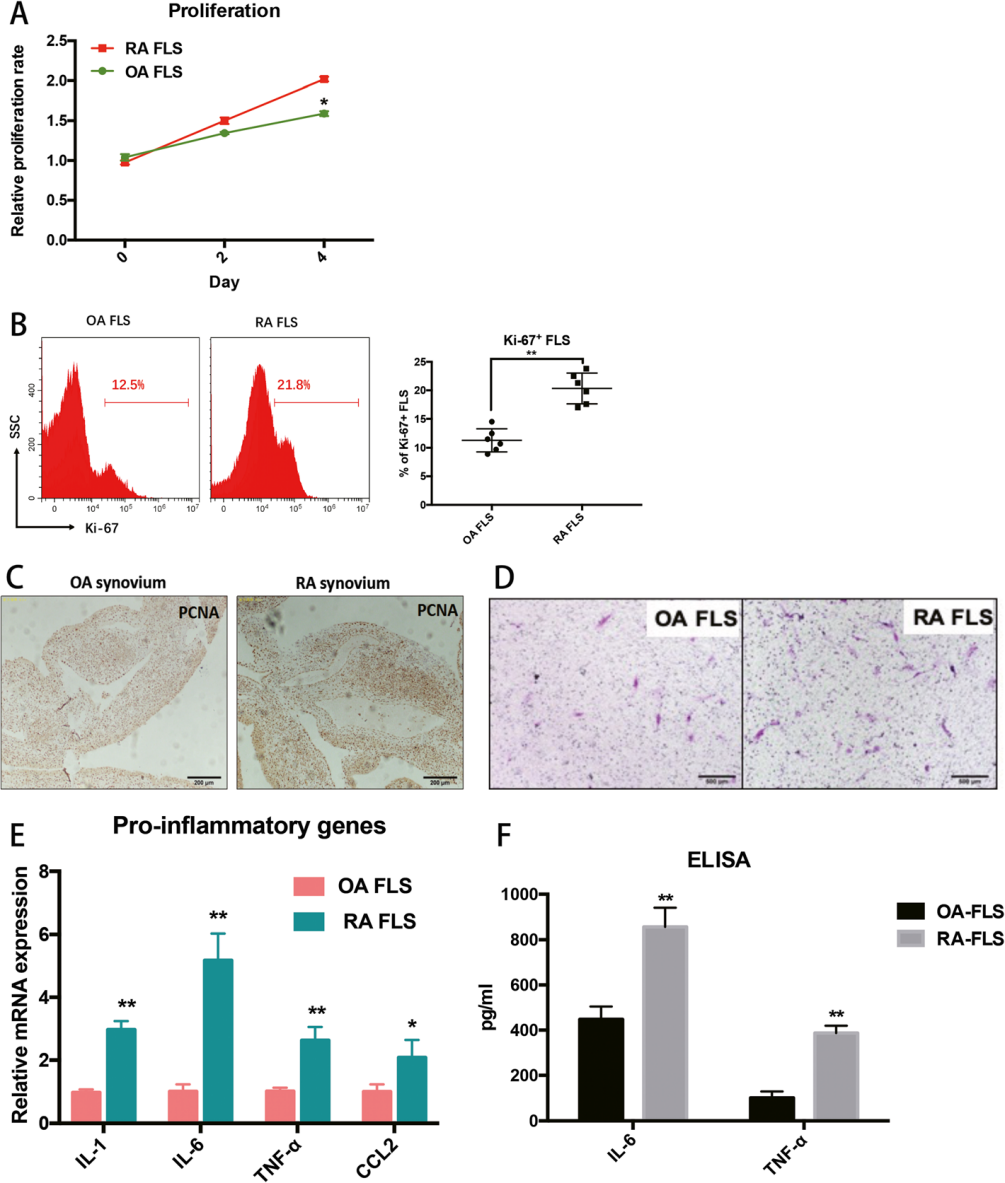
**a**-**b** The proliferative ability of OA and RA FLS was evaluated by CCK-8 assay and Ki-67 staining. **c** PCNA staining in OA and RA synovial tissues further validated that the percent of infiltrated PCNA^+^ cells was higher in RA synovial tissues compared to OA tissues. **d** RA FLS showed a more aggressive migrating feature when compared to OA FLS. **e** Expression of inflammatory genes, IL-1, IL-6, TNF-α and CCL2, are more highly expressed in RA FLS than in OA FLS. **f** Two pro-inflammatory cytokines, IL-6 and TNF-α, were significantly up-regulated in cell culture supernatant from RA FLS. Each bar in the figure represents the mean ± SEM of triplicates. *P < 0.05 and ** *P* < 0.01

Hyperplastic FLS are a key feature of arthritis, especially RA. Therefore, the proliferative ability of OA and RA FLS were initially evaluated by CCK-8 assay and Ki-67 staining. The results showed that RA FLS had a more rapid proliferative ability than OA FLS (Fig. 3a and b). PCNA staining in OA and RA synovial tissues further validated that the percentage of infiltrated PCNA^+^ cells was higher in RA synovial tissues than in an OA synovial tissues (Fig. 3c). In addition, examination of invasive behaviour in FLS revealed that both OA and RA FLS migrated through the Matrigel-coated insert, and RA FLS showed a more aggressive migrating nature than the OA FLS (Fig. 3d).

Analysis of gene expression in cells by qRT-PCR identified mRNA expression for inflammatory IL-1, IL-6, TNF-α and CCL2 markers in the FLS from 6 OA and RA patients. All inflammatory genes were more highly expressed in RA FLS than they were in OA FLS (Fig. 3e). Moreover, supernatants from OA and RA FLS were also collected to analyse pro-inflammatory cytokine production. The results demonstrated that two pro-inflammatory cytokines, IL-6 and TNF-α, were significantly up-regulated in the culture supernatant from RA FLS (Fig. 3f), suggesting that RA FLS show more inflammatory characteristics than do OA FLS.

**Fig. 4:**
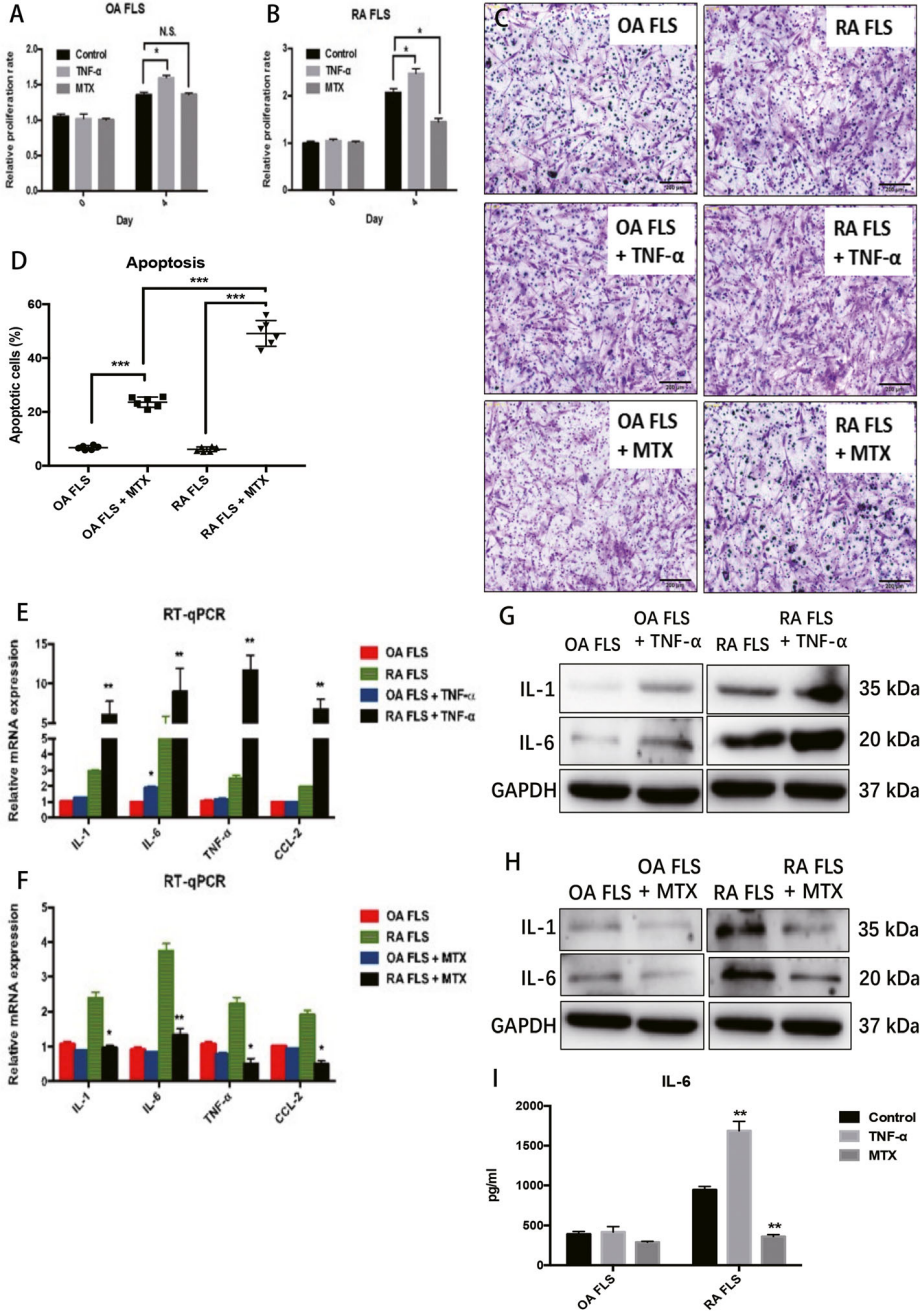
The effects of TNF-α and MTX on OA FLS and RA FLS. **a**-**b** The effects of TNF-α and MTX on the proliferation of OA FLS and RA FLS. **c** The effects of TNF-α and MTX on the migration of OA FLS and RA FLS. **d** The effects of MTX on apoptosis of OA FLS and RA FLS. **e**-**f** The effects of TNF-α and MTX on the mRNA expression of pro-inflammatory genes in OA FLS and RA FLS. **e**-**f** The effects of TNF-α and MTX on the protein expression of IL-1 and IL-6 in OA FLS and RA FLS. **i** The effects of TNF-α and MTX on IL-6 production from OA FLS and RA FLS. Each bar in the figure represents the mean ± SEM of triplicates. Scale bar=500 μm. *P < 0.05 and ** *P* < 0.01

Moreover, OA and RA FLS were examined in response to treatment with the pro-inflammatory cytokine TNF-α or the anti-inflammatory drug MTX to determine the inflammatory phenotype these cells. Treatment with TNF-α (20 ng/mL) resulted in a significant increase in the proliferation of both OA and RA FLS, and the inductive effect in RA FLS was greater (Fig. 4a and b). Treatment with MTX (100 μM) resulted in a decrease in proliferation of RA FLS but did not show any repressive effect on OA FLS (Fig. 4a and b). In addition, Matrigel-treated transwells were also used to evaluate the effects of TNF-α or MTX on the invasive capabilities of OA FLS and RA FLS. The results showed that TNF-α or MTX treatments could induce and inhibit the invasive abilities of OA and RA FLS, respectively (Fig. 4c). Annexin V/PI staining was used to compare the effect of MTX on apoptosis in OA and RA FLS. MTX induced apoptosis of both OA and RA FLS, and the OA FLS were more sensitive to the apoptosis-inducing effects of MTX when compared to RA FLS (Fig. 4d). The pro-inflammatory gene expression in OA and RA FLS were also evaluated in TNF-α or MTX treatment conditions. Although TNF-α could induce all four genes in both OA and RA FLS, the inductive effects of TNF-α on RA FLS were much more obvious than they were in the OA FLS (Fig. 4e and f). Similarly, the response of RA FLS showed that they were more sensitive to the anti-inflammatory drug MTX than the OA FLS were (Fig. 4e and f). Western blot results validated the effects of TNF-α and MTX on the protein expression of IL-1 and IL-6 from OA and RA FLS in vitro (Fig. 4g and h). Accordingly, IL-6 is also significantly up-regulated in TNF-treated RA FLS (Fig. 4i). In addition, MTX could inhibit IL-6 production from OA and RA FLS. The inhibitory effect of MTX on IL-6 secretion is much greater in RA FLS.

## Discussion

For inflammatory joint diseases such as OA and RA, FLS are an essential part of inflammation and joint erosion [13]. Human FLS isolated from the synovial tissues of OA and RA patients has been extensively used in many studies. These cells have been well characterized in multiple disease states, including RA and OA [7, 14]. However, the isolation efficiency of FLS from these studies, which use methods such as the differential plating of dissected tissues or magnetic bead isolation, is quite low. Moreover, significant contamination in FLS primary cultures was also observed. Therefore, a strict sorting strategy was used in the current study to isolate FLS from OA and RA synovial tissues by removing the main contaminating cell types (macrophages and endothelial cells). We successfully isolated FLS from RA and OA synovial tissues by combining enzyme digestion (collagenase IV/ deoxyribonuclease I digestion and red cell lysis buffer/FcR blocker buffer) and following a stringent flow sorting method (CD45^−^CD31^−^CD146^−^CD235a^−^CD90^+^PDPN^+^). OA FLS and RA FLS were compared and characterized based on multiple criteria to provide optimal confirmation of their origin and purity. The markers used in the FLS sorting strategy included general stromal fibroblast markers such as PDPN and CD90 [15] and more specific FLS markers such as ICAM1 and VCAM1 [16]. The analysis of PDPN/CD90/VCAM1/ICAM1 expression allowed us to better understand and distinguish OA FLS and RA FLS.

In addition to surface markers of FLS, the present study also performed side-by-side comparisons of some basic cellular features of OA FLS and RA FLS, analysing proliferation, migration, expression/secretion of inflammatory cytokines, and response to pro-inflammatory cytokines and anti-inflammatory drugs. In general, RA FLS shows more aggressive cellular behaviour compared to OA FLS, including a more rapid proliferation rate, stronger invasive ability, and higher expression and secretion of inflammatory cytokines. These observations are consistent with our knowledge of the arthritis features exhibited by OA and RA. Moreover, higher expression of inflammatory markers, such as CCL2, IL-6, IL-1β and TNF-α, were also observed in RA FLS when compared to FLS isolated from the less inflamed OA synovium. OA FLS and RA FLS also show different responses to the pro-inflammatory cytokine TNF-α or the anti-inflammatory drug MTX. TNF-α could induce proliferation/migration of both OA FLS and RA FLS, while the inductive effect on proliferation was more obvious in RA FLS. In contrast, MTX could inhibit RA FLS proliferation but did not affect the proliferation of OA FLS in vitro. The different responses to MTX were further investigated with apoptosis experiments: MTX greatly induced apoptosis of RA FLS and induced less apoptosis in OA FLS. However, low concentrations of MTX (10 μM) only slightly promoted apoptosis of RA FLS and OA FLS (data not shown). The high dose of MTX can induce a series of severe side effects [17], which is one of the main drawbacks to the clinical use of MTX to treat RA. However, after TNF-α or MTX treatment, the amount of IL-6 secreted by RA FLS was significantly induced or repressed, respectively. However, TNF-α did not affect IL-6 secreted by OA FLS, and MTX only slightly decreased IL-6 production from OA FLS. These results suggest that the response to and the underlying mechanism for MTX is different between OA FLS and RA FLS.

MTX also inhibited up-regulated inflammatory markers [18]. TNF-α significantly induced RA FLS [19–21] and modulated a panel of pro-inflammatory cytokines that act as an essential promoter of inflammation [22]. TNF-α depleting antibodies have also been shown to be an effective treatment for RA. In response to TNF-α, some pro-inflammatory markers were greatly up-regulated. In the current study, RA FLS were more sensitive to TNF-α and MTX, suggesting that RA FLS have a more inflammatory status than OA FLS. In RA, FLS-secreted IL-6 could drive the differentiation of T helper (Th) 17 cells [5]. Our results confirmed that IL-6 was significantly induced in RA FLS at both the mRNA and protein levels. In addition, IL-6 was more sensitive to pro- (TNF-α) and anti-inflammatory (MTX) treatment; therefore, IL-6 is a valuable marker of inflammation in RA FLS. With the pro-inflammatory genes used in this study, IL-6 will provide a standard to compare and distinguish FLS biology to distinguish OA and RA patients.

Although these inflammatory genes and markers have been previously validated with mice FLS culture in vitro [11], it is unknown how the human FLS compare to FLS isolated from other arthritis models such as collagen antibody-induced arthritis (CIA) and antigen-induced arthritis (AA). Therefore, in the future, it will be of great interest to compare the cellular features of human FLS and arthritis mouse models.

In summary, we characterized FLS isolated by collagenase digestion of synovial tissues from OA and RA patients by using a strict sorting strategy. The main cellular features were compared between OA FLS and RA FLS here. The current report provides an isolation and characterization standard for future research on human OA and RA FLS.

## Conclusion

By using a stringent sorting strategy, we comprehensively characterized and compared the cellular function and inflammatory phenotype (proliferation, migration, apoptosis, expression and production of inflammatory markers and response to pro-inflammatory cytokines and anti-inflammatory drugs) of FLS from the synovium of OA and RA patients in vitro. The parallel comparison of OA FLS and RA FLS lays a foundation for when FLS are considered potential therapies for anti-inflammatory treatment of OA and RA.

## Additional file

**Additional file 1: Figure S1.** The isotype controls were included in the FACS detection for proteins with relatively low expression (IgG1-FTIC for CD146, and REA control-FITC for CD31 and CD235a).

## Data Availability

The raw data will be made available from the corresponding author upon reasonable request.

## Abbreviations

AA: Antigen-induced arthritis
ACR: American College of Rheumatology
APC: Allophycocyanin
CCK-8: Cell Counting Kit-8
CIA: Collagen antibody-induced arthritis
DAB: Diaminobenzadine
DMEM: Dulbecco’s Modified Eagle’s Medium
EULAR: European League Against Rheumatism
FACS: Fluorescence-activated cell sorting
FBS: Fetal bovine serum
FITC: Fluorescein isothiocyanate
FLS: Fibroblast-like synoviocytes
H&E: Hematoxylin-eosin
HRP: Horseradish peroxidase
IHC: Immunohistochemistry
IL-1β: Interleukin-1 beta
IL-6: Interleukin-6
MTX: Methotrexate
OA: Osteoarthritis
PBS: Phosphate buffered saline
PE: Phycoerythrin
PI: Propidium iodide
qRT-PCR: Quantitative real-time polymerase chain reaction
RA: Rheumatoid arthritis
SEM: Standard Error of Mean
TNF-α: Tumor necrosis factor-alpha
