# Supplementary figures and images for: Parallel comparison of fibroblast-like synoviocytes from the surgically removed hyperplastic synovial tissues of rheumatoid arthritis and osteoarthritis patients
Source: BMC Musculoskelet Disord. 2019 Dec 7;20:591. doi: 10.1186/s12891-019-2977-2 (PMC6898940; doi:10.1186/s12891-019-2977-2)

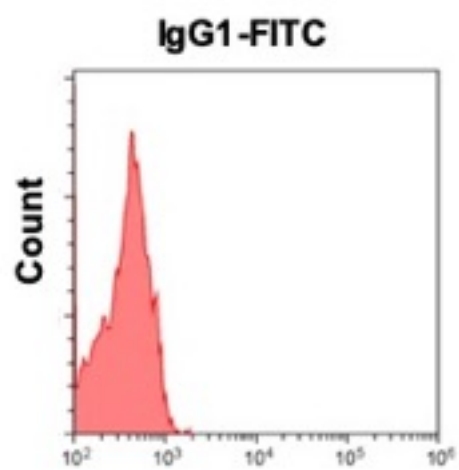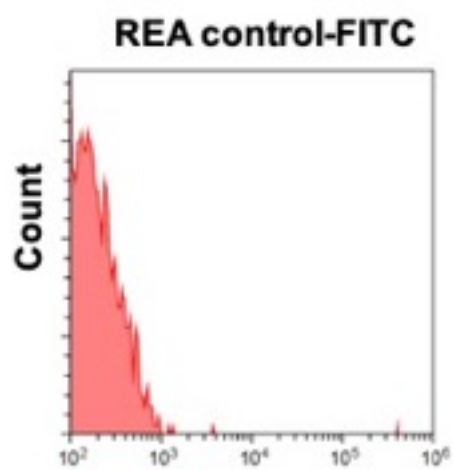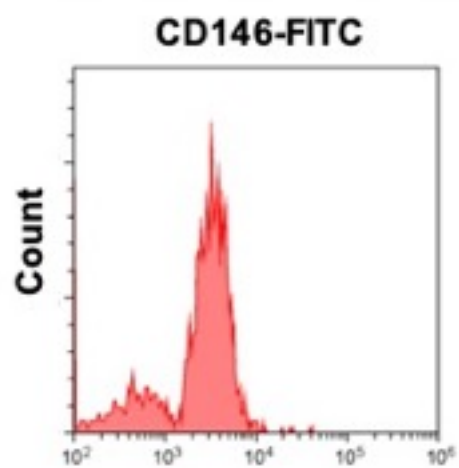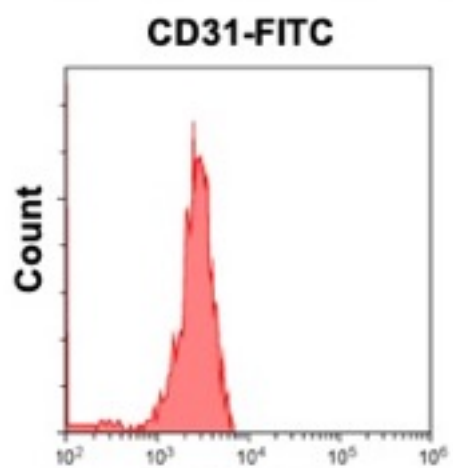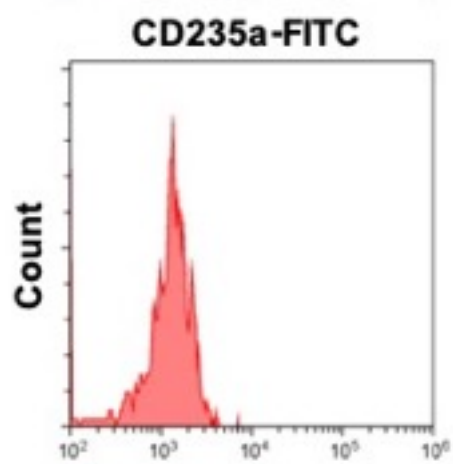

Supplement: Supplementary file 1 — Additional file 1: Figure S1. The isotype controls were included in the FACS detection for proteins with relatively low expression (IgG1-FTIC for CD146, and REA control-FITC for CD31 and CD235a). [file 12891_2019_2977_MOESM1_ESM.pdf]
